# The effect of hypertensive disorders in pregnancy on small for gestational age and stillbirth: a population based study

**DOI:** 10.1186/1471-2393-4-17

**Published:** 2004-08-06

**Authors:** Victoria M Allen, KS Joseph, Kellie E Murphy, Laura A Magee, Arne Ohlsson

**Affiliations:** 1 Department of Obstetrics and Gynaecology, Dalhousie University, Halifax, Nova Scotia, Canada; 2 Perinatal Epidemiology Research Unit, Department of Obstetrics and Gynaecology and of Pediatrics, Dalhousie University, Halifax, Nova Scotia, Canada; 3 Department of Obstetrics and Gynaecology, University of Toronto, Toronto, Ontario, Canada; 4 Department of Medicine, University of British Columbia, Vancouver, British Columbia, Canada; 5 Department of Pediatrics, University of Toronto, Toronto, Ontario, Canada

## Abstract

**Background:**

Hypertensive disorders in pregnancy are leading causes of maternal, fetal and neonatal morbidity and mortality worldwide. However, studies attempting to quantify the effect of hypertension on adverse perinatal outcomes have been mostly conducted in tertiary centres. This population-based study explored the frequency of hypertensive disorders in pregnancy and the associated increase in small for gestational age (SGA) and stillbirth.

**Methods:**

We used information on all pregnant women and births, in the Canadian province of Nova Scotia, between 1988 and 2000. Pregnancies were excluded if delivery occurred < 20 weeks, if birthweight was < 500 grams, if there was a high-order multiple pregnancy (greater than twin gestation), or a major fetal anomaly.

**Results:**

The study population included 135,466 pregnancies. Of these, 7.7% had mild pregnancy-induced hypertension (PIH), 1.3% had severe PIH, 0.2% had HELLP (hemolysis, elevated liver enzymes, low platelets), 0.02% had eclampsia, 0.6% had chronic hypertension, and 0.4% had chronic hypertension with superimposed PIH. Women with any hypertension in pregnancy were 1.6 (95% CI 1.5–1.6) times more likely to have a live birth with SGA and 1.4 (95% CI 1.1–1.8) times more likely to have a stillbirth as compared with normotensive women. Adjusted analyses showed that women with gestational hypertension without proteinuria (mild PIH) and with proteinuria (severe PIH, HELLP, or eclampsia) were more likely to have infants with SGA (RR 1.5, 95% CI 1.4–1.6 and RR 3.2, 95% CI 2.8–3.6, respectively). Women with pre-existing hypertension were also more likely to give birth to an infant with SGA (RR 2.5, 95% CI 2.2–3.0) or to have a stillbirth (RR 3.2, 95% CI 1.9–5.4).

**Conclusions:**

This large, population-based study confirms and quantifies the magnitude of the excess risk of small for gestational age and stillbirth among births to women with hypertensive disease in pregnancy.

## Background

Hypertensive disorders in pregnancy complicate approximately 10–16% of pregnancies and are leading causes of maternal, fetal and neonatal morbidity and mortality worldwide [1-3]. Definitions, classifications, assessment and management of hypertensive disorders vary considerably in the literature and from country to country [4]: thus, it is difficult to compare results from different studies. Past studies have attempted to quantify the effect of hypertension on adverse perinatal outcomes. To date, the majority of study designs have included retrospective and prospective cohort studies [5-9,12-20], as well as randomized-controlled trials that assessed the impact of antihypertensive medication on maternal and perinatal outcomes[10,11]. For the most part, these studies have been concentrated in tertiary referral centres, and suggest that hypertension in pregnancy leads to an increased risk of small for gestational age (SGA) and preterm birth.

We, therefore, carried out a population-based study to quantify the frequency of hypertensive disorders in pregnancy and also the excess risk of SGA and stillbirth that is associated with this pregnancy complication. We investigated the way in which SGA and stillbirth were modified by other factors that also have a serious influence on SGA and stillbirth, for example, whether a twin gestation modifies the effect of hypertension on SGA, or whether smoking modifies the effect of hypertension on stillbirth.

## Methods

### Population

The study population included all births to residents of the province of Nova Scotia, Canada between 1988 and 2000. Information on these births was obtained from the Nova Scotia Atlee Perinatal Database. The Nova Scotia Atlee Perinatal Database includes several hundred variables containing maternal and newborn information, such as demographic variables, procedures, interventions, maternal and newborn diagnoses and morbidity and mortality information for every pregnancy and birth occurring in Nova Scotia hospitals and to Nova Scotia residents since 1988.

Pregnancies were excluded if delivery occurred < 20 weeks, if birthweight was < 500 g, if there was a higher order pregnancy (greater than twin gestation), or a known major fetal anomaly. Information in the database on the type of hypertensive disorder is directly coded from medical charts. The diagnosis of hypertensive disorders in pregnancy was made if it occurred in the antepartum or postpartum period. Mild pregnancy induced hypertension (PIH) included physician-diagnosed mild pregnancy induced hypertension if in the chart, transient hypertension or a diastolic blood pressure exceeding 90 mmHg on two or more occasions in a 24-hour period. Severe pregnancy induced hypertension included physician-diagnosed severe pregnancy induced hypertension, diastolic blood pressure ≥ 110 mm Hg on at least two occasions within a 6-hour period, if magnesium sulfate was administered for seizure prophylaxis, if there was ≥ 2+ proteinuria, low platelets (<100,000), and/or elevated liver enzymes (ALT > 35 u/L, AST > 30 u/L and/or LDH > 670 u/L). HELLP syndrome (hemolysis, elevated liver enzymes, low platelets) included physician-diagnosed HELLP. Eclampsia included physician-diagnosed eclampsia or one or more convulsions not attributable to other cerebral conditions such as epilepsy or cerebral hemorrhage in a patient with hypertension. Chronic hypertensive disease included a history of hypertensive disease when not pregnant, prior to current pregnancy or prior to 20 weeks of the current pregnancy. Pregnancy induced hypertension superimposed on chronic hypertension included physician-diagnosed pregnancy induced hypertension superimposed on chronic hypertension or if there was hemolysis, elevated liver enzymes or low platelets.

We first examined the maternal characteristics of study subjects with hypertension in pregnancy. For this analysis, hypertensive disorders in pregnancy were defined according to Nova Scotia Atlee Perinatal Database definitions: mild PIH, severe PIH, HELLP, eclampsia, chronic hypertension and chronic hypertension with superimposed PIH. Maternal characteristics which were considered included age, marital status, parity, prepregnancy weight, pregnancy weight gain, administration of antenatal steroids, smoking, drug abuse, the presence of anemia (Hgb < 10 gm%), gestational diabetes (two abnormal values on a glucose tolerance test in pregnancy or if insulin was administered for the first time in pregnancy), pre-existing diabetes, and twin gestation.

For the multivariate analyses, these database definitions were grouped to more closely approximate commonly used definitions such as those proposed by the Canadian Hypertensive Society and other international organizations [4]. These groups were defined as gestational hypertension without proteinuria (including the database entity mild pregnancy induced hypertension), gestational hypertension with proteinuria (including the database entities severe pregnancy induced hypertension, HELLP, and eclampsia) and pre-existing hypertension (including the database entities chronic hypertension and chronic hypertension with superimposed pregnancy induced hypertension).

Only live births were considered in the analysis of small for gestational age, while all births were considered for the stillbirth analyses. Small for gestational age was defined as birthweight for gestational age that was less than the sex-specific 10^th ^percentile cut-off of a recently published Canadian fetal growth reference [21]. Stillbirth was defined as fetal death before birth, with gestational age ≥ 20 weeks and birthweight ≥ 500 grams.

Ethical approval for the study was obtained from the Research Ethics Boards at Dalhousie University in Halifax, Nova Scotia, the Reproductive Care Program of Nova Scotia and the IWK Health Centre in Halifax, Nova Scotia.

### Statistical analysis

Exact binomial 95% confidence intervals were calculated for rates of hypertensive disorders in pregnancy. Descriptive analyses were carried out on maternal data to ascertain the association between maternal characteristics and hypertensive disorders in pregnancy. Categorical data between hypertensive and normotensive pregnancies were compared using chi-square and Fisher's exact tests, where appropriate.

Logistic regression analyses were carried out to determine the adjusted odds ratios (OR) and 95% confidence intervals (CI) expressing the relationship between any hypertensive disorder in pregnancy and groups of hypertension (i.e., gestational hypertension without proteinuria, gestational hypertension with proteinuria, pre-existing hypertension) and the two dichotomous primary outcomes (SGA and stillbirth). Backward stepwise elimination of variables was carried out to identify all significant determinants of the outcome. Modification of the effect of hypertensive disorders on SGA or stillbirth was investigated, based on clinical understanding. Variables considered to be in the causal pathway between the determinant and the outcome (e.g., SGA in the analysis of stillbirth) were not adjusted for in the model [22-24]. When the outcome rate was low we assumed the odds ratio was equal to the relative risk, but not in situations when the outcome rate was high (>10 percent). The significance level selected was *P *< .05 and all tests were two-tailed. Statistical analyses were performed using the SAS programming package for Windows (Version 8.0).

## Results

### Frequency of hypertensive disease

The study population included 135,466 pregnancies. Of these, 7.7% (95% CI 7.6,7.9) had mild PIH, 1.3% (95% CI 1.3,1.4) had severe PIH, 0.2% (95% CI 0.1,0.2) had HELLP, 0.02% (95% CI 0.02,0.03) had eclampsia, 0.6% (95% CI 0.5,0.6) had chronic hypertension and 0.4% (95% CI 0.3,0.4) had chronic hypertension with superimposed PIH. The overall rate of hypertensive disease in pregnancy was 10.1% (95% CI 10.0,10.3).

### Maternal characteristics

Table 1 summarizes the characteristics of women with hypertensive disorders in pregnancy from 1988–2000. Women with severe PIH were less likely to be married (63.5%), while women with chronic hypertension (with or without superimposed PIH) were more likely to be married (83.2% and 77.3%, respectively) compared with normotensive women (69.6%). Women with hypertensive disorders were more likely to be nulliparous (range 42.2% to 78.2%) as compared with normotensive women (41.9%). Women with hypertensive disorders in pregnancy had higher pre-pregnancy weight (range 66.8 kg to 82.0 kg) compared with normotensive women (64.4 kg). While women with PIH (mild or severe) had on average a greater weight gain in pregnancy (16.2 kg and 16.5 kg, respectively), women with chronic hypertension (with or without superimposed PIH) had a lower weight gain (12.2 kg and 13.2 kg, respectively) compared with normotensive women (14.4 kg). A smaller proportion of women with hypertensive disorders in pregnancy smoked (range 13.0% to 22.7%) as compared with normotensive women (30.7%). Women with hypertensive disorders in pregnancy were more likely to have gestational diabetes (range 4.6% to 9.1%) and pre-existing diabetes (range 0.8% to 2.5%) compared with normotensive women (2.3% and 0.3%, respectively). Twin pregnancies were more likely to be complicated by hypertensive disorders (range 2.3% to 6.4%) as compared with normotensive pregnancies (1.0%).

**Table 1:** Characteristics of women with and without hypertensive disorders in pregnancy, Nova Scotia, 1988–2000.

Variable	Normotensive n = 121,760	Mild PIH n = 10,460	Severe PIH n = 1,770	HELLP n = 202	Eclampsia n = 32	Chronic HTN n = 767	Chronic HTN +PIH n = 475
Mean age in years (SD)	27.8 (5.3)	27.5 (5.4)*	26.9 (5.7)*	28.9 (5.5)*	25.6 (6.8)*	31.1 (5.0)*	30.5 (5.1)*
Married (%)	84,739 (69.6)	7,530 (72.0)	1,124 (63.5)*	145 (71.8)	18 (56.3)	638 (83.2)*	367 (77.3)*
Nulliparous (%)	51,057 (41.9)	6,760 (64.6)*	1,328 (75.0)*	158 (78.2)*	20 (62.5)*	324 (42.2)*	259 (54.5)*
Mean prepregnancy weight in kg (SD)	64.4 (13.8)	70.6 (16.7)*	67.6 (15.6)*	66.8 (16.0)*	68.2 (16.9)	82.0 (20.9)*	81.3 (20.9)*
Mean weight gain in kg (SD)	14.4 (6.0)	16.2 (6.9)*	16.5 (6.9)*	14.8 (7.0)	16.2 (6.2)	12.2 (6.7)*	13.2 (7.3)*
Smokes any cigarettes (%)	35,846 (30.7)	2,157 (21.5)*	376 (22.7)*	31 (16.0)*	6 (21.4)	150 (20.4)*	59 (13.0)*
Anemia (%)	3,441 (2.8)	242 (2.3)*	78 (4.3)*	15 (7.4)*	0 (0)	31 (4.1)	20 (4.2)
Gestational Diabetes (%)	2,749 (2.3)	485 (4.6)*	84 (4.8)*	3 (1.5)	2 (6.3)	70 (9.1)*	34 (7.2)*
Preexisting Diabetes (%)	331 (0.3)	85 (0.8)*	29 (1.6)*	4 (2.0)*	0 (0)	19 (2.5)*	9 (1.9)*
Anti-hypertensive medication (%)	0 (0)	107 (1.0)	97 (5.5)	23 (11.4)	5 (15.6)	146 (19.0)	122 (25.7)
Twins (%)	1,231 (1.0)	244 (2.3)*	60 (3.4)*	13 (6.4)*	0 (0)	7 (0.9)	13 (2.7)*

PIH denotes pregnancy induced hypertension, HELLP denotes hemolysis, elevated liver enzymes, low platelets syndrome, HTN denotes hypertension. * Denotes hypertension categories significantly different from the normotensive category.

### Small for gestational age

Table 2 summarizes the crude relationships between hypertension in pregnancy and SGA. There was an increased risk of SGA among infants born to women with any hypertensive disorder (RR 1.6, 95% CI 1.5,1.6) compared with infants born to women with normotensive pregnancies. There was an increased risk of SGA among infants born to women with mild PIH (RR 1.3, 95% CI 1.3,1.4), severe PIH (RR 2.5, 95% CI 2.3,2.8), HELLP (RR 3.8, 95% CI 3.2,4.5), eclampsia (RR 3.5, 95% CI 2.2,5.7), chronic hypertension (RR 1.4, 95% CI 1.1,1.6) and chronic hypertension with PIH (RR 2.2, 95% CI 1.8,2.6) compared with infants born to women with normotensive pregnancies.

**Table 2:** Comparison of small for gestational age (SGA, <10^th ^percentile) rates among live births to hypertensive vs. normotensive women, Nova Scotia, 1988–2000.

	Total No.	SGA		Relative Risk	95% CI	*P *value
		No.	%			
Normotensive women	122,394	12,032	9.8	1.0	-	-
All hypertensive women	13,940	2,131	15.3	1.6	1.5,1.6	<0.001
Mild PIH	10,639	1,384	13.0	1.3	1.3,1.4	<0.001
Severe PIH	1,814	453	25.0	2.5	2.3,2.8	<0.001
HELLP	212	79	37.3	3.8	3.2,4.5	<0.001
Eclampsia	32	11	34.4	3.5	2.2,5.7	<0.001
Chronic Hypertension	766	102	13.3	1.4	1.1,1.6	0.002
Chronic hypertension and PIH	477	102	21.4	2.2	1.8,2.6	<0.001

PIH denotes pregnancy induced hypertension, HELLP denotes hemolysis, elevated liver enzymes, low platelets syndrome, HTN denotes hypertension, CI denotes Confidence interval.

After controlling for potential confounders, women with any hypertensive disorder were 1.8 (95% CI 1.7,1.9, *P *< .001) times more likely to have a live birth with SGA as compared with normotensive women. Women with gestational hypertension without proteinuria were 1.5 (95% CI 1.4,1.6, *P *< .001) times more likely to have a live birth with SGA as compared with normotensive women. Similarly, women with gestational hypertension with proteinuria were 3.3 (95% CI 3.0,3.9, *P *< .001) times more likely and women with pre-existing hypertension were 2.5 (95% CI 2.1,2.9, *P *< .001) times more likely to have a live birth with SGA as compared with normotensive women.

### Stillbirth

Table 3 summarizes the crude relationships between hypertension in pregnancy and stillbirth. There was an increased risk of stillbirth among women with any hypertensive disorder (RR 1.4, 95% CI 1.1,1.8) and among women with pregnancies complicated by chronic hypertension (RR 2.4, 95% CI 1.2,5.1) or chronic hypertension with superimposed PIH (RR 4.4, 95% CI 2.2,8.8), compared with normotensive pregnancies.

**Table 3:** Comparison of stillbirth rates among all births to hypertensive vs. normotensive women, Nova Scotia, 1988–2000.

	Total No.	Stillbirths		Relative Risk	95% CI	*P *value
		No.	%			
Normotensive women	122,855	461	0.4	1.0	-	-
All hypertensive women	14,013	73	0.52	1.4	1.1,1.8	0.01
Mild PIH	10,683	44	0.4	1.1	0.8,1.5	0.55
Severe PIH	1,826	12	0.7	1.8	1.0,3.1	0.50
HELLP	214	2	0.9	2.5	0.6,9.9	0.19
Eclampsia	32	0	0.0	-	0.0,41.0	1.00
Chronic Hypertension	773	7	0.9	2.4	1.2,5.1	0.03
Chronic hypertension and PIH	485	8	1.7	4.4	2.2,8.8	<0.001

PIH denotes pregnancy induced hypertension, HELLP denotes hemolysis, elevated liver enzymes, low platelets syndrome, HTN denotes hypertension, CI denotes Confidence interval.

After controlling for potential confounders, women with any hypertensive disorder were 1.4 (95% CI 1.1,1.8, *P *= .02) times more likely to have a stillbirth as compared with normotensive women (Table 4). Women with pre-existing hypertension were 3.2 (95% CI 1.9,5.4, *P *< .001) times more likely to have a stillbirth as compared with normotensive women.

**Table 4:** Effect of hypertensive disorders in pregnancy on small for gestational age (< 10^th ^percentile) and stillbirth, Nova Scotia, 1988–2000.

	Crude			Adjusted		
	Odds ratio	95% CI	*P *value	Odds ratio	95% CI	*P *value
Small for gestational age*						
Normotensive women	1.0	-	-	1.0	-	-
Hypertensive women (any type)	1.6	1.5,1.6	<0.001	1.8	1.7,1.9	<0.001
Gestational hypertension without proteinuria	1.3	1.3,1.4	<0.001	1.5	1.4,1.6	<0.001
Gestational hypertension withproteinuria	2.7	2.5,2.9	<0.001	3.3	3.0,3.9	<0.001
Pre-existing hypertension	1.7	1.5,1.9	<0.001	2.5	2.1,2.9	<0.001
Stillbirth**						
Normotensive women	1.0	-	-	1.0	-	-
Hypertensive women (any type)	1.4	1.1,1.8	0.01	1.4	1.1,1.8	0.02
Gestational hypertension without proteinuria	1.1	0.8,1.5	0.55	1.1	0.8,1.5	0.60
Gestational hypertension with proteinuria	1.8	1.0,3.1	0.03	1.6	0.9,2.9	0.08
Pre-existing hypertension	3.2	1.9,5.3	<0.001	3.2	1.9,5.4	<0.001

CI denotes Confidence interval. * Adjusted for smoking, maternal age, gestational diabetes, pre-existing diabetes, maternal anemia, nulliparity, marital status, drug abuse, prepregnancy weight, weight gain, antenatal steroids, twins and infant sex. ** Adjusted for smoking, maternal autoantibodies, maternal age, pre-existing diabetes, maternal anemia,prepregnancy weight and twins.

Twin pregnancy modified the effect of gestational hypertension with or without proteinuria on small for gestational age (Table 5). A woman with a singleton pregnancy who had gestational hypertension without proteinuria had a 1.5 fold increase in risk of SGA compared with a woman with a normotensive singleton pregnancy. A woman with a twin pregnancy had a 4.7 fold increase in risk of SGA, but the woman with gestational hypertension without proteinuria and a twin pregnancy had a less than expected increase in risk of SGA (1.5 × 4.7 × 0.7 = 4.9 as opposed to 1.5 × 4.7 = 7.1) i.e., the combined effect of a twin pregnancy and gestational hypertension without proteinuria was less than what would be expected under a multiplicative (logistic) model. Similarly, a woman with gestational hypertension with proteinuria and a twin pregnancy had a less than expected increase in risk of SGA (5.2 compared to 17.4). A woman with pre-existing hypertension who smoked had a less than expected increase in risk of SGA (4.4 compared to 7.3). A woman with gestational hypertension without proteinuria and who smoked had a greater than expected increase in risk of stillbirth (2.8 compared to 1.1).

**Table 5:** Effect of hypertensive disorders in pregnancy on small for gestational age (< 10^th ^percentile) and stillbirth, with modeling of effect-modification by twin pregnancy and maternal smoking, Nova Scotia, 1988–2000.

	Adjusted		
	Odds ratio	95% CI	*P *value
Small for gestational age			
Normotensive women	1.0	-	-
Gestational hypertension without proteinuria	1.5	1.4,1.6	<0.001
Gestational hypertension with proteinuria	3.7	3.3,4.1	<0.001
Pre-existing hypertension	2.5	2.1,3.0	<0.001
Twins	4.7	4.3,5.2	<0.001
Gestational hypertension without proteinuria × twins	0.7	0.6,0.9	0.01
Gestational hypertension with proteinuria × twins	0.3	0.2,0.4	<0.001
Pre-existing hypertension × twins	0.7	0.3,1.4	0.29
Smoking	2.9	2.8,3.0	<0.001
Gestational hypertension without proteinuria × smoking	1.0	0.9,1.2	0.96
Gestational hypertension with proteinuria × smoking	0.9	0.7,1.1	0.28
Pre-existing hypertension × smoking	0.6	0.4,0.8	0.002
Stillbirth			
Normotensive women	1.0	-	-
Gestational hypertension without proteinuria	0.8	0.5,1.2	0.24
Gestational hypertension with proteinuria	1.4	0.6,2.9	0.42
Pre-existing hypertension	3.5	2.0,6.2	<0.001
Smoking	1.4	1.2,1.7	<0.001
Gestational hypertension without proteinuria × smoking	2.5	1.3,4.7	0.006
Gestational hypertension with proteinuria × smoking	1.8	0.4,5.3	0.33
Pre-existing hypertension × smoking	0.6	0.1,2.5	0.43

CI denotes Confidence interval.** Adjusted for smoking, maternal age, gestational diabetes, pre-existing diabetes,maternal anemia, nulliparity, marital status, drug abuse, prepregnancy weight,weight gain, antenatal steroids, twins and infant sex. ** Adjusted for smoking, maternal autoantibodies, maternal age, pre-existing diabetes, maternal anemia, prepregnancy weight and twins.

## Discussion

This large population-based cohort study examined the magnitude of the risks of small for gestational age and stillbirth in women with hypertension in pregnancy. Hypertensive disorders in pregnancy had a significant effect on rates of SGA after adjusting for potential confounders. Pre-existing hypertension had a significant effect on stillbirth rates after adjusting for potential confounders. Modification of the effect of hypertension on SGA and stillbirth was observed among women who also had a twin pregnancy or were also smokers.

The rate of hypertensive disorders in pregnancy in this population (10.1%, 95% CI 10.0,10.3) was similar to that reported in the literature (10–16%). The rate of mild PIH in this population was 7.7%, compared with 6–7% in the literature. The rate of severe PIH (1.3%), HELLP (0.2%) and eclampsia (0.02%) were lower than expected (5–6%). The rate of pre-existing hypertension (0.6%) and pre-existing hypertension with superimposed PIH (0.4%) were lower than expected (3–5% and 0.8–1.3%, respectively) [25,26]. These differences in rates from what is expected from the published literature may be explained by the fact that most previous studies were conducted in high-risk populations in referral hospitals. Differences between our study findings and those from other population-based studies [12-20] may be due to potential differences in population characteristics, and clinical practice factors including quality of diagnostic information. Our study also identified maternal characteristics previously known to be associated with hypertensive disorders in pregnancy, including nulliparity [25], older age [27], diabetes [28], twin pregnancy [29] and smoking [30].

Small for gestational age is a more appropriate measure of fetal growth than birthweight alone. Our study evaluated birthweight for gestational age and gender among live births to normotensive and hypertensive pregnancies using a recent population-based Canadian reference [21]. Women with any hypertensive disorder, gestational hypertension with or without proteinuria, and pre-existing hypertension were at a significantly higher risk of having a SGA infant (relative risk 1.8, 1.5, 3.3, and 2.5, respectively). The risk of SGA was higher among hypertensive women with a twin pregnancy. Similar patterns in risk for SGA have been seen in other populations [13,14,16]. Women with any hypertensive disorder and pre-existing hypertension were at significantly higher risk of stillbirth compared with women having normotensive pregnancies (RR 1.4 and 3.2, respectively). Similarities in risk for fetal mortality between women with gestational hypertension with or without proteinuria and women who were normotensive in pregnancy have been reported elsewhere [31].

Chance and confounding are unlikely explanations for the results of our study, because of the large study size, and adjustment for potential confounders using logistic regression. This study was not able to correct for the degree of blood pressure control, which may have an effect on fetal growth. While retrospective studies in general are limited by the reliability of data, information in the Nova Scotia Atlee Perinatal Database is of high quality. Routine data checks and edits are made at the time of data collection, and validation [32] and reabstraction studies attest to the quality of the data in this large clinical database. Our study was limited by the definitions for hypertensive disorders in pregnancy used by the Nova Scotia Atlee Perinatal Database, and while these definitions are not exactly the same as commonly used definitions, they approximate definitions proposed by the Canadian Hypertension Society and other organizations [4]. The number of comparisons carried out requires that *P *values associated with the results be interpreted with caution.

## Conclusions

This population-based cohort study demonstrates that women with hypertensive disease in pregnancy are at significantly higher risk of having pregnancies complicated by small for gestational age (<10^th ^percentile) and stillbirth in comparison with women with normotensive pregnancies. Twin gestation and smoking were important modifiers of the effect of hypertension on SGA and stillbirth. This study allowed the quantification of risks of adverse outcomes in women with pregnancies complicated by hypertension, confirming associations in the published literature and allowing appropriate counseling and monitoring in the management of these women, as well as providing baseline risks which may be used in future intervention studies.

## Competing interests

None declared.

## Authors' contributions

VMA proposed the study, carried out the preliminary analyses and wrote the paper. All authors discussed the analyses, contributed to the intellectual content of the paper and approved the final version. VMA and KEM provided the maternal-fetal medicine perspective, KSJ provided the general medical and epidemiologic input, LAM provided the general and obstetrical medicine perspective, and AO provided the neonatal-perinatal perspective and epidemiologic input.

## Pre-publication history

The pre-publication history for this paper can be accessed here:


